# An Automated Sprinkler Cooling System Effectively Alleviates Heat Stress in Dairy Cows

**DOI:** 10.3390/ani14172586

**Published:** 2024-09-05

**Authors:** En Liu, Liping Liu, Zhili Zhang, Mingren Qu, Fuguang Xue

**Affiliations:** 1 Jiangxi Province Key Laboratory of Animal Nutrition, Engineering Research Center of Feed Development, Jiangxi Agricultural University, Nanchang 330029, China; liuenvip@163.com; 2 School of Food Engineering, Anhui College of Science and Technology, Chuzhou 233100, China; liuliping321407@163.com; 3 Modern Farming (Wuhe) Co., Ltd., Bengbu 233311, China; zhangzlds@163.com

**Keywords:** heat stress, automatic spraying, rumen fermentation, milk production, rumen microbiota

## Abstract

**Simple Summary:**

Heat stress detrimentally affects dairy cows, resulting in economic losses during dairy production. Therefore, this study aimed to evaluate the effects of an automatic spraying method on alleviating heat stress in cows and identifying potential mechanisms. Our findings showed that the automated sprinkler cooling system treatment effectively reduced body temperatures, improved milk yield and quality, enhanced rumen fermentability, modulated rumen microbial communities, and significantly proliferated carbohydrate-degrading bacteria. Moreover, our study demonstrated that the automatic spraying cooling system had modulatory effects on rumen microbiota composition and fermentation function, and causatively ameliorated the side effects of heat stress in dairy cows.

**Abstract:**

(1) Background: Heat stress detrimentally restricted economic growth in dairy production. In particular, the cooling mechanism of the spraying system effectively reduced both environmental and shell temperatures. This study was designed to investigate the underlying modulatory mechanism of an automatic cooling system in alleviating heat-stressed dairy cows. (2) Methods: A total of 1208 multiparous dairy cows was randomly allocated into six barns, three of which were equipped with automatic sprinklers (SPs), while the other three were considered the controls (CONs). Each barn was considered a replicate. (3) Results: Body temperatures and milk somatic cell counts significantly decreased, while DMI, milk yield, and milk fat content significantly increased under SP treatment. Rumen fermentability was enhanced, embodied by the increased levels of total VFA, acetate, propionate, and butyrate after SP treatment. The rumen microbiota results showed the relative abundances of fiber-degrading bacteria, including the *Fibrobacters*, *Saccharofermentans, Lachnospira, Pseudobutyrivibrio*, *Selenomonas,* and *Succinivibrio*, which significantly increased after receiving the SP treatment. (4) Conclusions: This study demonstrated that SP effectively alleviated heat stress and improved production performances and milk quality through modulating the rumen microbiota composition and fermentation function of dairy cows.

## 1. Introduction

The average surface temperature has been increasing by 0.19 °C per decade [1]. Consequently, the frequency and duration of extreme weather events, especially heatwaves and extreme precipitation, have significantly increased [2]. As the ruminal fermentation process produces excessive heat, the mammary gland conducts high anabolic activities, and cows have a low surface-area-to-mass ratio, making lactating cows more vulnerable to heat stress [3,4]. It has been reported that, when the temperature–humidity index (THI) reached 72 (recent research indicated this value can be as low as 68 or 70), side effects of heat stress on dairy cows notably appeared [5,6]. The detrimental impacts of heat stress on dairy cows mainly include reduced feed intake, milk production metrics, milk protein metrics, and the following conception rate [7], as well as an increased risk of ruminal acidosis and enteric methane (CH4) production [8]. Therefore, heat stress is an obvious restrictive factor in dairy cow production and will worsen with the continuously increasing global temperature. Thus, determining an effective cooling method to attenuate heat stress in cows is of the utmost importance.

Re-establishing the physiological thermal energy balance between heat acquisition (e.g., maintenance, exercise, growth, lactation, gestation, and feed intake) and dissipation in the environment is critical for attenuating heat stress [9,10]. During the production process, several cooling options, such as shading and air fans, can be used to lower temperatures and restore the normal physiology of cows [11]. Furthermore, nutritional strategies, such as supplementing appropriate energy ingredients, minerals, vitamins, antioxidants, prebiotics, and probiotics, have also been applied to ameliorate heat stress [12]. Compared with nutritional regulations, directly cooling the environment (e.g., cooling with sprinklers) appears to be the most economical and efficient method for relieving heat stress [13]. Cooling with sprinklers has been proven to be successful in reducing shell and body temperatures, increasing heat evaporation to a lower respiration rate, enhancing feed intake, and promoting digestibility [14], which contributed to increased milk and milk protein production [15,16,17,18]. However, the mechanisms underlying these positive effects in attenuating heat stress have not been elucidated.

Ruminal microbiota is of vital importance for animal health and production performance, as microorganisms can ferment feed ingredients to supply critical metabolites for the host [19,20,21,22,23,24,25]. For example, ruminal microbial communities can produce nutritional fermentation metabolites, like vitamins and functional fatty acids [19,26,27,28,29]. These metabolites can be adsorbed through rumen epithelial cells and then transported through the blood to the target tissues to regulate tissue functions. Recent research has documented that rumen microbial communities are directly regulated by heat stress [30,31,32,33]. For instance, Zhao et al. (2019) found that heat stress led to ruminal bacterial composition alteration and functional deterioration, increased lactate, and reduced acetate-producing bacterial relative abundance [30]. Other studies have disclosed that ruminal fiber-degrading bacteria populations (e.g., *Fibrobacter*) decreased, while starch-degrading bacteria populations (e.g., *Clostridium* and *Streptococcus*) increased [34,35] in response to heat stress. Another study found that live yeast supplementation positively ameliorated heat stress in dairy cows through regulating the microbiota composition and rumen fermentation in the rumen and hindgut [36]. These studies demonstrate that ruminal microbiota response may be an underlying mechanism of the positive effects on heat stress in ruminal animals.

Therefore, the present study evaluated the effect of an automatic sprinkler on the production performances of cows and elucidated the potential mechanism through considering the ruminal microbiota.

## 2. Materials and Methods

### 2.1. Experiment Animals and Management

**Figure 1:**
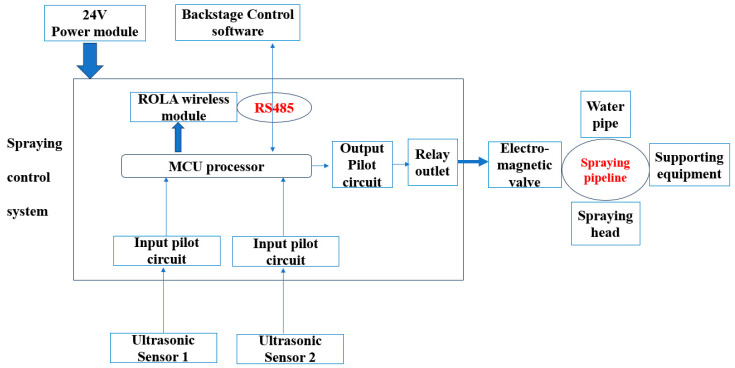
Schematic diagram showing the mechanism of the sprinkler. MCU = microprogramed control unit. The MCU processor controls the ultrasonic sensors to transmit detection signals through a pulse width modulation (PMW). ROLA = reliability optimum link allocation. The MCU controls ultrasonic sensors 1 and 2 to send detection signals, followed by the acquirement of detection distance, and determines the detected distance between the sensors after receiving the backward signal at 100 m. The MCU controls the initiation and turnoff mechanisms of the sprinkler and compares the ultrasonic detection distance acquired using the above method with the set control parameters to determine whether the cow entered the detection area.

The experiment was conducted in the Bengbu dairy farm, Modern Farming (Wuhe) Co. Ltd., Anhui Province, China (32.92 N, 117.38 E), from 10 June 2023, to 10 August 2023. A total of 1208 multiparous Chinese Holstein dairy cows with an average live weight of 683.6 ± 27.3 kg, lactation of 199.3 ± 16.8 d, and lactating parities of 2.88 ± 0.49 was used and randomly allocated to 6 barns. Three barns were equipped with automatic sprinklers (SPs), and each barn was considered a replicate. The other three barns followed the routine feeding procedure (CON) without sprinklers. The schematic diagram of the sprinkler is shown in Figure 1.

All cows were reared in a 312 m long × 96 m wide shed to ensure the same feeding environment. Diets were formulated, according to NRC (2001) [37], to meet the energy requirements of Holstein dairy cows, yielding 30 kg of milk/day with 3.5% milk fat and 3.0% true protein. The nutrient level and ingredient composition of the employed diet are shown in Table 1. Cows were fed three times per day, at 06:00, 13:00, and 21:00. During the experimental period, all cows had free access to food and water. Temperature and humidity were recorded every day, and THIs were calculated using the following equation, as previously reported in [38]: THI = (1.8 × T + 32) − [(0.55 − 0.0055 × RH) × (1.8 × T −26)], where T = temperature and RH = relative humidity. When the THI ≥ 70, heat stress occurred in high-yielding dairy cows [6]. Cows were milked three times per day (08:00, 14:00, and 20:00 h) [39].

### 2.2. Feed Intake and Composition Analysis

The average daily intake in barns was determined based on the dry matter intake (DMI), which was calculated as the difference between the feed offered and the residues on the dry matter basis. Feed samples were collected from each feeding time and mixed for analysis. Air-dried samples were obtained from the fresh feed and dried using a forced-air oven (GZX-9246MBE, Shanghai Boxunshiye Co., Ltd., Shanghai, China) at 65 °C for 48 h. Then, the absolute dried feed samples were obtained from the air-dried samples and dried further at 105 °C for 3 h using the forced-air oven.

The net energy (NE) level of the feed was calculated using the methodology in [40]. The feed compositions were determined according to the AOAC (2007) method. A Kjeldahl nitrogen analyzer (SKD-1800, Shanghai Peiou Analytical Instrument Co., Ltd., Shanghai, China) was used to determine the crude protein (CP) level. The ether extract (EE) was determined using the Soxhlet extractor and calculated with the following formula:

EE = [(m3 − m2)/m1] × f

where m1 = the mass of the sample in grams (g); m2 = the mass of the flask with emery in grams (g); m3 = the mass of the flask containing emery and the obtained dry residue of petroleum ether extraction in grams (g); and f = the unit of the correction factor in grams per kilogram (g/kg) (f = 1000 g/kg).

A semi-automatic fiber analyzer (A200i, ANKOM, Macedon, NY, USA) was used to determine the neutral detergent fiber (NDF) and acid detergent fiber (ADF) levels. Calcium (Ca) and phosphorus (P) levels were determined using the near-infrared spectroscopy (NIRS) method (NIRS DS2500 analyzer, FOSS Co., Ltd., DK-3400 Hilleroed, Denmark).

### 2.3. Milk Production and Composition

Daily milk yield was automatically recorded through the rotary milking facilities (9JRP-50P2100, Delaval, Israel). Milk samples were collected from each treatment during the last three consecutive days and stored in 100 mL vials with 2-bromo-2-nitropropan-1,3-diol at 4 °C for subsequent analysis.

Milk protein and fat were measured using a near-infrared analyzer (MilkoScanTM 7 RM, Foss Electric, Denmark). The somatic cell count (SCC) was measured using an SCC rapid analyzer (Fossomatic 7/7 DC, FOSS Co., Ltd., DK-3400 Hilleroed, Denmark).

### 2.4. Body Temperature Measurement

The body temperature of 30 cows in each treatment was measured once a week via the rectal thermometry method using a thermometer (VT 1831; Microlife AG Espenstrasse 139, CH-9443 Widnau, Switzerland).

### 2.5. Rumen Content Collection and Fermentation Parameter Analysis

Three cows from each barn with similar body weights (693.6 ± 12.3 kg), for a total of nine cows, were selected from the CON and SP groups for sample collection during the second middle lactation period. Three hours after morning feeding, 100 mL of rumen contents from 18 dairy cows was collected using an esophageal tube on the last day of the experiment [41]. The first 200 mL contents was discarded to avoid potential saliva contamination. All rumen samples were divided into two parts. One part was analyzed for pH, volatile fatty acid (VFA), and ammonia-N (NH_3_-N). The pH value of each rumen fluid sample was measured immediately using a portable pH meter (Testo 205, Testo AG, Lenzkirch, Germany). Individual and total VFAs (TVFAs) in the aliquots of ruminal fluid were determined using a gas chromatograph (GC-2010, Shimadzu, Kyoto, Japan). NH3-N concentration was determined using the indophenol method, and the absorbance value was measured using a UV-2600 ultraviolet spectrophotometer (Tianmei Ltd., Chaoyang, Beijing, China) [42]. The other part was rapidly frozen with liquid nitrogen and then stored at −80 °C for further analysis.

### 2.6. Rumen Microbial Communities Measurement

Rumen microbial DNA was extracted from approximately 1.0 mL of rumen content using the MagBind^®^ Soil DNA Kit (M5636, Omega, Norcross, GA, USA). DNA concentration, purity, and quality were assessed using a spectrophotometer and agarose gel electrophoresis. The V4 and V3 regions of the 16S rRNA gene were amplified using universal primers (F: ACTCCTACGGGAGGCAGCAG and R: GGACTACHVGGGTWTCTAAT). The PCR product mixture was purified with a Qiagen Gel Extraction Kit (Qiagen, Hilden, Germany). Sequencing was conducted on an Illumina MiSeq PE300 platform /NovaSeq PE250 platform (Illumina, San Diego, CA, USA) in a commercial laboratory. The quality filtering of raw tags was performed under standard filtering conditions to obtain high-quality clean tags, according to the Quantitative Insights into Microbial Ecology (QIIME, V1.7.0, San Diego, CA, USA, V1.7.0) quality control process. Sequences within similarity >97% were assigned to the same operational taxonomic unit (OTU). For each representative sequence, the SILVA classifier algorithm was used to annotate the taxonomic information from the GreenGene Database. Then, the species abundances and α- and β-diversity indices were analyzed at different taxonomic levels.

### 2.7. Statistical Analysis

A normal distribution test was first conducted on the production performances, milk quality, rumen fermentable parameters, and relative abundances of rumen microbial communities using the SAS (Statistics Analysis System, version 9.2, SAS Institute Inc., Cary, NC, USA) procedure, denoted as “proc univariate data=test normal”. Data were presented as mean ± SE. Further differential analysis on the above-mentioned parameters was performed using an unpaired two-tailed Student’s T-test. *p*-value < 0.05 was significant, and 0.05 ≤ *p* ≤ 0.10 indicated a trend.

## 3. Results

### 3.1. Effects of Automatic Spraying on Body Temperature, Milk Yield and Content, and Pregnancy Rate under Heat Stress Conditions

**Figure 2:**
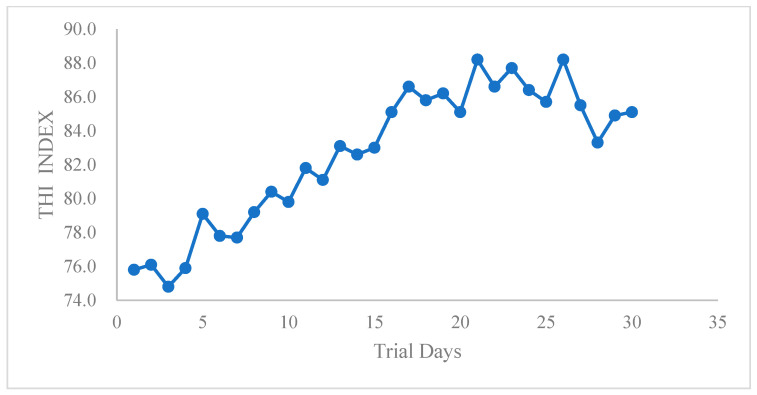
THI record during the heat stress trial.

In this study, the temperature and relative humidity of the feeding lair were recorded at five different locations, and the THI was recorded throughout the entire experiment (Figure 2). During the experimental period, environmental THI exceeded 74, indicating that heat stress occurred throughout the whole experiment.

The body temperature and production performances of dairy cows receiving the automatic spraying and control treatments were measured, as shown in Table 2. The body temperature of cows that received SP treatment significantly decreased (*p* < 0.05), indicating that cooling was successfully achieved. In addition, DMI and milk yield significantly increased (*p* < 0.05) after spraying treatment under the heat stress condition, which demonstrated that automatic spraying effectively ameliorated the detrimental effects of heat stress on cows. Moreover, the milk fat content demonstrated a tendency to increase (*p* = 0.056). Although not significant, milk protein levels were slightly elevated (*p* = 0.412). SCC was significantly decreased in the SP group (*p* < 0.05). Collectively, these data suggest that automatic spraying effectively alleviates the adverse effects of heat stress on cows, improving their lactating performance and pregnancy rates.

### 3.2. Effects of Automatic Spraying on Rumen Fermentable Parameters under the Heat Stress Condition

Rumen fermentation parameters, including pH, NH_3_-N, and VFAs, are presented in Table 3. Automatic spraying had no effect on the rumen pH and NH_3_-N levels (*p* > 0.05) but significantly increased rumen acetate, isobutyrate, and butyrate levels (*p* < 0.05), as well as propionate levels (*p* = 0.054), in the SP group compared to the CON group. Furthermore, we consistently noted that the TVFA level was significantly higher in the SP group than the CON group (*p* < 0.05). However, the acetic-to-propionic ratio remained unchanged between both groups (*p* > 0.05). Collectively, these data suggest that automatic spraying effectively enhances rumen fermentation functions.

### 3.3. Effects of Automatic Spraying on Rumen Microbiome

We investigated the modulatory effects of the automatic spraying treatment on the rumen microbial composition. The results of rumen microbiota sequencing show that a total of 5800 OTUs, 17 phyla, and 290 genera was identified after quality control, as shown in Table S1.

All identified bacteria were chosen for further α-diversity parameter analysis of ruminal bacteria between the SP and CON groups, and the results are shown in Table 4. The ACE index and observed species were significantly increased in the SP group compared with the CON group (*p* < 0.05), and a similar increase was also observed for the Chao1 index (*p* = 0.062). No other significant alterations were observed between the SP and CON groups.

**Figure 3:**
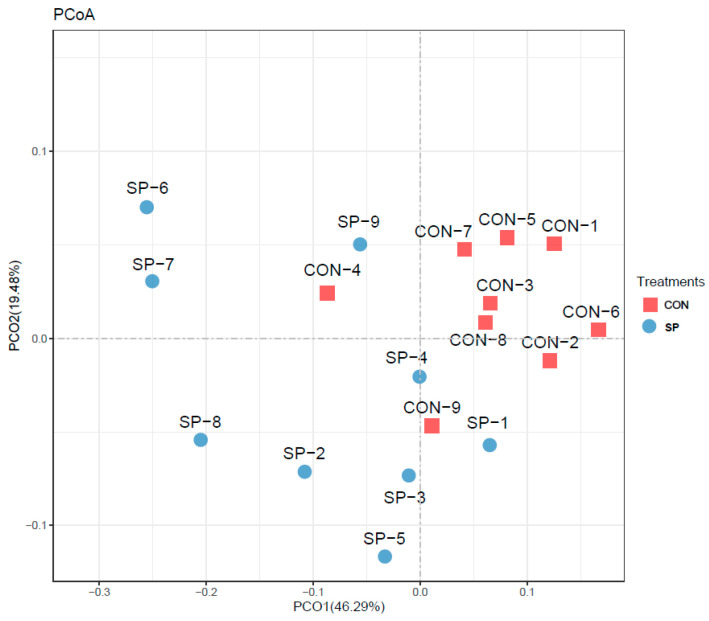
Principal coordinate analysis (PCoA) of community structures of the rumen microbiota after sprinkling treatment. CON = control treatment; SP = sprinkling treatment.

Then, PCoA analysis was performed to assess β-diversity. The PCoA results clarify the monolithic discrepancy in the microbial profiles between the SP and CON groups. As shown in Figure 3, PCoA axes 1 and 2 account for 46.29% and 19.48%, respectively. Bacterial communities in the SP group could be separated from those in the CON group, indicating that automatic spraying modulated the rumen microbiota composition under the heat stress condition.

At the phylum level, *Firmicutes*, *Bacteroidetes*, and *Tenericutes* were the most abundant microorganisms identified in the rumen under the heat stress condition (Table 5). The relative abundance of *Fibrobacters* and *Bacteroidetes* in the rumen significantly increased in the SP group compared to the CON group. 

As Table 6 shows, genera of *Prevotella*, *Ruminococcaceae*, *Succiniclasticum*, *Lachnospiraceae*, and *Eubacterium* supported the top-five most abundant bacterial communities in both the SP and CON groups. The relative abundance of *Succiniclasticum*, *Butyrivibrio*, *Pseudobutyrivibrio*, *Bifidobacterium*, and *Streptococcus* significantly increased (*p* < 0.05) in the SP group compared with the CON group. In contrast, the relative abundance of *Ruminococcaceae* and *Succinivibrio* significantly decreased (*p* < 0.05).

**Figure 4:**
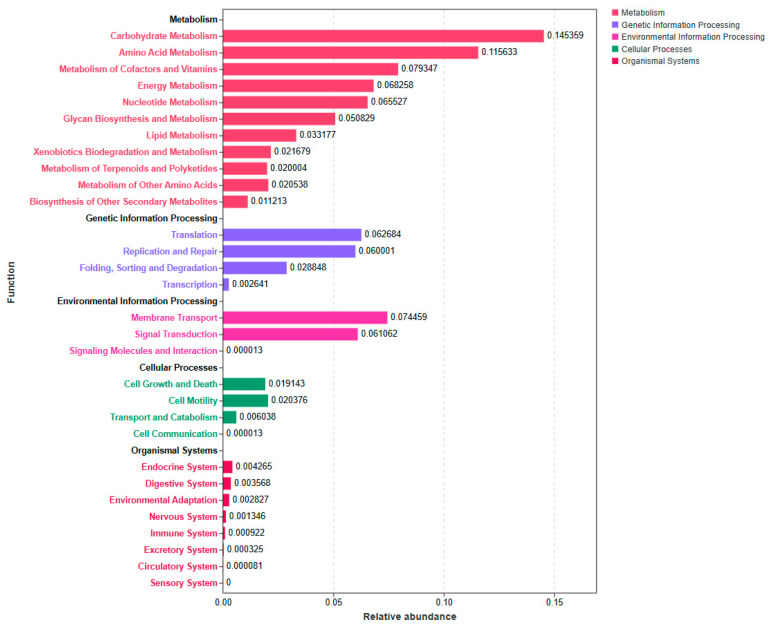
Functional prediction analysis of the significantly altered bacterial communities between the SP and CON groups in dairy cows using Tax4Fun. CON = control treatment; SP = sprinkling treatment.

Functions that potentially presented in the differentially identified microbiota were predicted using Tax4Fun, and the results are shown in Figure 4. Metabolic processes, including carbohydrate, amino acid, and energy metabolisms, and other cofactors, and genetic information processing, including the translation, replication, and repair processes, were the predominant functional pathways. In particular, the functions of differentially abundant bacteria were mostly enriched in carbohydrate, amino acid, energy, cofactor, and vitamin metabolisms. In contrast, lower relative abundances were observed in lipid metabolism and secondary metabolites. Genetic information processing methods, including the translation, replication, and repair processes, and environmental information processing methods, including membrane transport and signal transduction, were also enriched via the differential abundant microbiota.

## 4. Discussion

Heat stress detrimentally influences dairy production. When heat stress occurs, dairy cows present reduced feed intake, dairy production, and milk quality; increased evaporated water loss; and metabolic disturbance. During the experiments, the THI remained over 72, indicating that cows were all heat stressed. Under heat stress conditions, high environmental temperatures improve body temperatures, which extends the feed duration in rumen and, in turn, activates the rumen sensor as the stomach expands [43]. This alteration impacted the hypothalamic anorexia center, resulting in a lowered appetite and feed intake [44]. As a result, DMI and energy intake also reduced. The automatic spray cooling method reduced cow body temperatures and SCC; increased DMI, milk yield and fat, and ICR; and enhanced rumen fermentation functions, effectively alleviating heat stress in cows.

Rumen microbial communities are critical to dairy health and production performances. One important function of rumen microorganisms is to provide the host with energy and functional metabolites through the metabolism of nutrient residues. Carbohydrates are usually converted to pyruvate and acetyl-CoA in the rumen via microorganisms through the glycolytic and pentose phosphate pathways, and finally metabolized to VFAs—especially acetate and propionate—to provide energy for production. In our present study, carbohydrate-degrading bacteria, such as *Pseudobutyrivibrio, Bifidobacterium,* and *Succinivibrio*, significantly increased after SP treatment, which may further indicate a higher energy provision for milk production and more physiological activities. It has been reported that approximately 70% to 80% of the energy absorbed by cows is provided via rumen fermentation processes [19]. Acetate was further transported into the mammary gland and synthesized into milk fat, while butyrate was utilized in the intestinal tract for epithelial development. In the present study, VFAs, including acetate, propionate, and butyrate, significantly increased in rumen fluids under SP treatment. TVFAs, especially the VFA composition and proportions, reflect energy balance and utilization in cows and act as effective indicators for rumen fermentation capacity [45]. In particular, acetate undergoes lipid synthesis in adipose and the mammary tissues of ruminants with a primary carbon source [46], which partially accounts for the increased milk fat level. In addition, heat stress triggered Toll-like receptor pathways and caused inflammatory responses. Deteriorated inflammation will cause mastitis and lead to increased milk SCC [47]. Butyrate serves as critical immune regulator, helpful in inhibiting mastitis [48], which may explain the reduced SCC in milk when the butyrate content increased. It is worth noting that, although rumen fermentation was enhanced via automatic spraying, the rumen acetate-to-propionate ratio did not differ between the SP and CON groups, suggesting that the rumen fermentation pattern remained unchanged. Therefore, further investigation is needed.

Ruminal homeostasis provided an ideal environment for nutrient digestion and transportation, energy generation, and microbial proliferation [49,50]. The rumen microbial composition and function significantly altered under heat-stressed environments, potentially disrupting primary homeostasis and further causing reduced feed intake and milk production. Rumen microbial richness and composition were modulated via automatic spraying, indicating the rumen microbial may contribute to the positive effects of automatic spraying under heat stress conditions. Zhao et al. (2019) and Uyeno et al. (2010) reported that the relative abundances of *Spirochaeta*, *Streptococcus*, and *Ruminobacter* increased and acetic acid decreased in the heat stress condition [30,51]. In agreement with these data, we found that the relevant abundance of *Streptococcus* decreased and the rumen acetic acid level increased. Under ruminal conditions, acetate was transported through the rumen epithelium and then utilized by mammary gland cells as the key substrate for milk fat synthesis. Therefore, milk fat content increased after SP treatment. In addition, some functional microorganism abundances were modulated via the automatic spraying treatment. For example, the relative abundances of *Prevotella* and *Pseudobutyrivibrio* increased, where *Prevotella* is important in starch and *Pseudobutyrivibrio* is a stressor-related genera. Indeed, these changes reduced fiber degradation and enhanced starch degradation [34,35], which ensured the abundant energy supply for body production and thus enhanced yield production.

## 5. Conclusions

This investigation showed that the automated sprinkler spray cooling treatment effectively alleviated heat stress in dairy cows through reducing body temperature, moderating microenvironmental conditions and humidity, and improving milk production. The rumen microbiota analysis indicated that the spray cooling treatment on dairy cows may also have modulatory effects on rumen microbes and fermentation functions, enhancing carbohydrate degradation in heat-stressed cows.

## Figures and Tables

**Table 1:** Diet nutrient level and ingredients (dry matter basis).

Items	Content
Ingredients (%)	
Corn	17.7
Corn silage	24.5
Soybean meal	12.3
Cottonseed meal	3.3
Pressure corn piece	8.2
Leymus chinensis	10.2
Distiller’s dried grains with soluble (DDGS)	3.1
Alfalfa hay	14.3
Beet pulp	4.8
Premix ^(1)^	1.0
NaCl	0.6
Total	100
Chemical composition	
NE ^(2)^ (MJ/kg)	7.13
EE (%)	4.56
CP (%)	17.36
ADF (%)	18.52
NDF (%)	31.34
Ca (%)	0.68
P (%)	0.41

^(1)^ One kilogram of the premix contained the following: Fe, 1400 mg; Cu, 1200 mg; Mn, 2400 mg; Zn, 5500 mg; Se, 40 mg; Co, 30 mg; I, 90 mg; VA, 900,000 IU; VD, 700,000 IU; VE, 9000 IU. ^(2)^ NE is a calculated value, whereas the other nutrients are measured values.

**Table 2:** Effects of sprinkling on production performances and meat quality during heat stress conditions.

Items	SP (*n* = 9)	CON (*n* = 9)	SE	*p*-Value
Body temperature	38.6	38.9	0.10	0.046
DMI	23.3	21.4	0.68	0.043
Milk yield	31.3	29.4	0.56	0.046
Milk fat	3.76	3.63	0.11	0.056
Milk protein	3.37	3.34	0.03	0.412
SCC	13.77	19.39	2.46	0.033

CON = control treatment; SP = sprinkling treatment. DMI = dry matter intake; SCC = somatic cell count; SE = standard error.

**Table 3:** Effects of sprinkling on rumen fermentable parameters during heat stress conditions.

Items	SP (*n* = 9)	CON (*n* = 9)	SE	*p*-Value
Rumen pH	6.08	6.14	0.08	0.351
NH_3_-N	17.73	15.43	1.53	0.168
Acetate	67.67	59.29	3.97	0.047
Propionate	23.79	20.44	1.65	0.054
Isobutyrate	1.17	0.65	0.16	0.004
Butyrate	16.52	14.25	1.08	0.049
Valerate	1.95	2.07	0.37	0.744
Isovalerate	2.40	2.44	0.34	0.915
TVFA	113.51	99.15	6.88	0.035
A:P	2.88	2.92	0.09	0.622

CON = control treatment, SP = sprinkling treatment, and SE = standard error.

**Table 4:** Effects of sprinkling on rumen bacteria α-diversity parameters during heat stress conditions.

Items	SP (*n* = 9)	CON (*n* = 9)	SE	*p*-Value
Shannon	7.85	7.66	0.10	0.182
Simpson	0.98	0.98	0.00	0.342
Ace	2356.5	2171.4	52.0	0.018
Chao1	2256.3	2116.6	51.2	0.062
observed_species	1934.9	1772.5	44.8	0.017

CON = control treatment, SP = sprinkling treatment, and SE = standard error.

**Table 5:** Effects of sprinkling on rumen bacteria diversities during heat stress conditions (level of phyla).

Items	SP (*n* = 9)	CON (*n* = 9)	SE	*p*-Value
*p__Actinobacteria*	6.47	5.51	0.76	0.229
*p__Fibrobacteres*	1.48	1.20	0.10	0.086
*p__Firmicutes*	15.44	15.35	0.11	0.940
*p__Bacteroidetes*	13.57	13.17	0.20	0.078
*p__Tenericutes*	8.96	8.55	0.23	0.101
*p__Cyanobacteria*	2.88	2.15	0.74	0.335
*p__Patescibacteria*	5.50	4.88	0.53	0.266
*p__Proteobacteria*	5.94	5.67	0.45	0.564
*p__Spirochaetes*	8.00	7.36	0.40	0.133
Others	5.82	5.20	0.42	0.122

CON = control treatment, SP = sprinkling treatment, and SE = standard error.

**Table 6:** Effects of sprinkling on relative rumen bacteria diversities during heat stress conditions (level of genera).

Items	SP (*n* = 9)	CON (*n* = 9)	SE	*p*-Value
*g__Prevotella*	18.66	16.75	1.239	0.064
*g__Ruminococcaceae*	15.64	20.20	0.189	0.005
*g__Succiniclasticum*	11.42	9.76	0.321	0.029
*g__Lachnospiraceae*	5.38	5.41	0.282	0.623
*g__Eubacterium*	4.49	4.07	0.206	0.141
*g__Ruminococcus*	4.37	4.95	0.256	0.015
*g__Shuttleworthia*	1.95	1.37	0.182	0.272
*g__Prevotellaceae*	1.28	1.25	0.237	0.371
*g__Acetitomaculum*	0.992	0.741	0.121	0.063
*g__Lachnoclostridium*	0.713	0.751	0.012	0.137
*g__Butyrivibrio*	0.534	0.418	0.034	0.035
*g__Ruminiclostridium*	0.258	0.284	0.041	0.108
*g__Pseudobutyrivibrio*	0.253	0.182	0.014	0.009
*g__Selenomonas*	0.074	0.041	0.021	0.167
*g__Lactobacillus*	0.069	0.061	0.010	0.089
*g__Bifidobacterium*	0.036	0.021	0.013	0.048
*g__Escherichia-Shigella*	0.024	0.033	0.008	0.271
*g__Bacteroides*	0.022	0.024	0.006	0.345
*g__Succinivibrio*	0.014	0.071	0.012	0.033
*g__Streptococcus*	0.019	0.014	0.003	0.029
*g__Butyricicoccus*	0.011	0.011	0.006	0.132
others	34.06	33.64	3.346	0.421

CON = control treatment, SP = sprinkling treatment, and SE = standard error.

## Data Availability

The data presented in the study are deposited in the NCBI Sequence Read Archive (SRA, http://www.ncbi.nlm.nih.gov/Traces/sra/, accessed on 10 June 2024), accession number PRJNA753017.

## Supplementary Materials

The following supporting information can be downloaded at: https://www.mdpi.com/article/10.3390/ani14172586/s1, Table S1: 16S rRNA results of rumen microbiota.
